# Gradual loss of mobile genetic elements in *Staphylococcus aureus* USA300 in a closed hospital niche

**DOI:** 10.1093/ismeco/ycaf105

**Published:** 2025-06-26

**Authors:** Anne-Gaelle Ranc, Patricia Martins Simões, Benjamin Youenou, Camille Kolenda, Céline Dupieux, Frédéric Laurent, Jean Philippe Rasigade, Anne Tristan, François Vandenesch

**Affiliations:** Centre National de Référence des Staphylocoques, Institut des agents infectieux, Hospices Civils de Lyon, F-69004 Lyon, France; Centre National de Référence des Staphylocoques, Institut des agents infectieux, Hospices Civils de Lyon, F-69004 Lyon, France; CIRI, Center for Integrative Research in Infectious diseases and Immunology, Université de Lyon, Inserm U1111, Université Claude Bernard Lyon 1, CNRS UMR5308, ENS de Lyon, F-69007 Lyon, France; Centre National de Référence des Staphylocoques, Institut des agents infectieux, Hospices Civils de Lyon, F-69004 Lyon, France; CIRI, Center for Integrative Research in Infectious diseases and Immunology, Université de Lyon, Inserm U1111, Université Claude Bernard Lyon 1, CNRS UMR5308, ENS de Lyon, F-69007 Lyon, France; Centre National de Référence des Staphylocoques, Institut des agents infectieux, Hospices Civils de Lyon, F-69004 Lyon, France; CIRI, Center for Integrative Research in Infectious diseases and Immunology, Université de Lyon, Inserm U1111, Université Claude Bernard Lyon 1, CNRS UMR5308, ENS de Lyon, F-69007 Lyon, France; Centre National de Référence des Staphylocoques, Institut des agents infectieux, Hospices Civils de Lyon, F-69004 Lyon, France; CIRI, Center for Integrative Research in Infectious diseases and Immunology, Université de Lyon, Inserm U1111, Université Claude Bernard Lyon 1, CNRS UMR5308, ENS de Lyon, F-69007 Lyon, France; Centre National de Référence des Staphylocoques, Institut des agents infectieux, Hospices Civils de Lyon, F-69004 Lyon, France; CIRI, Center for Integrative Research in Infectious diseases and Immunology, Université de Lyon, Inserm U1111, Université Claude Bernard Lyon 1, CNRS UMR5308, ENS de Lyon, F-69007 Lyon, France; CIRI, Center for Integrative Research in Infectious diseases and Immunology, Université de Lyon, Inserm U1111, Université Claude Bernard Lyon 1, CNRS UMR5308, ENS de Lyon, F-69007 Lyon, France; Centre National de Référence des Staphylocoques, Institut des agents infectieux, Hospices Civils de Lyon, F-69004 Lyon, France; CIRI, Center for Integrative Research in Infectious diseases and Immunology, Université de Lyon, Inserm U1111, Université Claude Bernard Lyon 1, CNRS UMR5308, ENS de Lyon, F-69007 Lyon, France; Centre National de Référence des Staphylocoques, Institut des agents infectieux, Hospices Civils de Lyon, F-69004 Lyon, France; CIRI, Center for Integrative Research in Infectious diseases and Immunology, Université de Lyon, Inserm U1111, Université Claude Bernard Lyon 1, CNRS UMR5308, ENS de Lyon, F-69007 Lyon, France

**Keywords:** *Staphylococcus aureus*mobile genetic elements, USA300 lineage

## Abstract

Our French National Reference Centre for Staphylococci was requested to determine the epidemiological link between 19 methicillin-susceptible/resistant Staphylococcus aureus (MSSA/MRSA) isolates obtained from patients and a healthcare worker in a long-term care hospital, following the death of a nurse from pneumonia presumed to be associated with the drainage of a patient with an active skin infection. Whole genome sequencing was performed on all isolates to characterize their virulome, resistome, and phylogenetic relationships. Phylogenetic analysis revealed that 12 isolates belonged to the North American USA300 lineage, which is the predominant MRSA in North America but is less prevalent in Europe. USA300 strains are typically described as belonging to the clonal complex 8 (CC8) and possessing several mobile genetic elements (MGEs): the pathogenicity island SaPI5, the PVL-encoding bacteriophage ϕSa2USA, the staphylococcal chromosomal cassette *mec*IVa (SCC*mec*IVa), and the arginine catabolic mobile element (ACME). All 12 isolates lacked SaPI5, and four possessed the other typical MGEs of the USA300 lineage. However, one isolate did not carry SCC*mec*IVa, six did not have ACME, and two did not carry ϕSa2USA. The topology of the phylogenetic tree and the phylodynamic analysis suggested the loss of SaPI5 before entry in the long-term hospital, which may have occurred 3–4 years before the current outbreak. As long-term hospital may represent a relatively closed and stable ecosystem, we concluded that this loss of MGEs is a phenomenon of genetic diversification driven by niche specialization, in this case, originally constituted by a healthcare institution.

## Brief communication

The emergence of the Staphylococcus aureus USA300 clade as the predominant methicillin-resistant S. aureus (MRSA) in both community and healthcare settings in North America and northern South America over the past two decades highlights its adaptation to a wide variety of clinical environments. First identified in a state prison outbreak in 1999, USA300 had become the most commonly isolated MRSA strain in the United States by 2011 [1–3]. The typical North American USA300 clone (NAE) is characterized by specific mobile genetic elements (MGEs): the pathogenicity island SaPI5, which encodes the staphylococcal enterotoxins *sek* and *seq*, the ϕSa2USA phage encoding the Panton-Valentine leukocidin (PVL) genes that impact the severity of community-acquired pneumonia [4], the staphylococcal chromosomal cassette SCC*mec*IVa harboring the methicillin resistance gene *mec*A, and the arginine catabolic mobile element (ACME) conferring competitive advantage in skin colonization and invasion [5, 6]. The close epidemiological Latin American USA300 variant (SAE) differs from North American USA300 in two key molecular features: the SCC*mec*IVc rather than the SCC*mec*IVa, and the copper and mercury resistance (COMER) element rather than ACME [7–10]. These mobile elements are considered essential to the evolutionary success of the USA300 NAE and SAE lineages, but strains lacking either of these elements have been observed [11–13]. The present study aimed to explore the transmission chain within a long-term care hospital following the death of a nurse due to necrotizing pneumonia presumed to be associated with the drainage of a patient with an active MRSA infection. This investigation has led us to explore the evolution of a USA300 clade with, in an unexpected and very original way, multiple MGE losses over a long dissemination period within a single institution.

**Figure 1:**
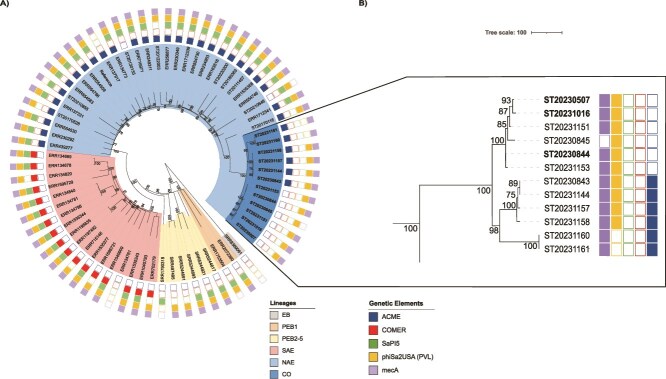
(A) Maximum likelihood tree of 69 CC8 *S. aureus* genomes including 21 ST8 methicillin-resistant or methicillin susceptible *S. aureus* (MRSA/MSSA) isolates from the French National Reference Center for Staphylococci (FNRCS) (12 from the current outbreak, CO; branches in bold) and 48 well-characterized USA300 North American Epidemics (NAE; *n* = 21), South American Epidemics (SAE; *n* = 18), pre-epidemic branches (PEB1, PEB2–5; *n* = 2 and 6, respectively), and early branching (EB; *n* = 1) genomes from Bianco *et al* [12]. The tree was constructed from the concatenated core-genome SNP sequences obtained after recombination event correction using gubbins v3.3 (3422 bp SNP alignment). The reference strain for alignment and SNP detection was the *S. aureus* reference strain USA300_THC1516 (GenBank accession number CP000730). Color codes used in the different rings are shown on the left; presence or absence of key genetic elements such as the arginine catabolic mobile element (ACME), the copper and mercury resistance (COMER) element, the pathogenicity island SaPI5, the ϕSa2USA phage encoding the Panton-Valentine leukocidin (PVL), and the methicillin resistance gene *mecA* are indicated with solid or hollow squares. (B) Maximum likelihood tree of the 12 MRSA/MSSA CC8 genomes from the current outbreak. Isolates highlighted in bold were isolated from the nurse (ST20230507) and the two potential source patients (ST20230844 and ST20231016). Color codes for MGEs as in Panel A. Support at nodes greater than 70 is indicated for both trees.

A total of 19 *S. aureus* isolates were sent to the French National Reference Centre for Staphylococci (FNRCS) for expert analysis between 11 February and 13 April 2023. One isolate was collected from the sputum of a nurse; the remaining isolates were obtained from a site of colonization or from the site of infection in patients on the same ward (Supplementary Table S1). All isolates underwent WGS. Libraries were prepared using the DNA Prep library kit and sequenced on the NextSeq 550 system (Illumina, USA) to obtain 150 bp paired-end reads. The trimmed reads were then employed to generate assemblies using SPAdes v3.14 [14]. The virulome and resistome were determined using an internal database (which contains most of the *S. aureus* virulence factors, such as all the leukocidins, exfoliative toxins, superantigenic toxins, capsule-associated genes, and immune invasion cluster genes), and AMRFinder, respectively. Core-genome SNPs were detected after alignment of trimmed reads on the chromosome of the S. aureus reference strain USA300_THC1516 (GenBank accession number CP000730) using snippy v4.6.0 (https://github.com/tseemann/snippy), excluding all positions with less than 10-fold sequencing depth and less than 90% unambiguous variant calls, and purged of potential recombination events using gubbins v3.3 [15]. Multilocus sequence typing (MLST) was carried out from the assemblies using mlst (https://github.com/tseemann/mlst) and the PubMLST database (https://pubmlst.org). Phylogenetic reconstruction was performed using IQTree [16] with the GTR Gamma model, including the ST8 isolates from this study, and 57 USA300 isolates chosen to account for the geographic and temporal diversity of this lineage including its different subclades and the presence/absence of the various MGEs. This collection was composed of nine USA300 isolates from the FNRCS collection, and 48 WGS data of previously published S. aureus strains [12].

**Figure 2:**
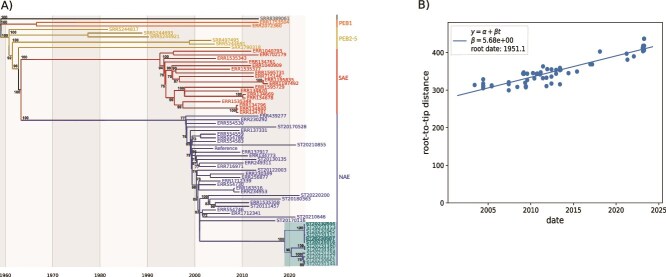
(A) Maximum likelihood time tree estimated from the initial tree topology, tip dates, and the core genome alignment purged of recombination events. Current outbreak (CO) isolates are highlighted in cyan. (B) The linear root-to-tip (RTT) regression (regression of tip times *vs* phylogenetic distances from root) allowing for the optimal root of the maximum-likelihood time tree in (A). A significant correlation was observed, indicating that the USA300 subset is a measurably evolving population.

Of the 19 isolates to be assessed, 12 were typed as ST8 with 11 MRSA (assessed by the presence of *mec*A) and 1 MSSA (lack of *mec*A). The seven remaining isolates belonged to ST5-MRSA (*n* = 3), ST5-MSSA (*n* = 1), and ST398-MSSA (*n* = 3); they did not possess the ϕSa2USA PVL phage. Phylogenetic analysis of the 12 ST8 isolates confirmed that they belonged to the NAE USA300 clade (Fig. 1). While the 12 isolates lacked SaPI5, four harbored the other MGEs typically associated with this lineage (SCC*mec*IVa, ACME, ϕSa2USA) and lacked COMER. However, one isolate lacked SCC*mec*IVa, six did not possess the ACME element, and two did not carry ϕSa2USA. The hypothesis deduced from the topology of the phylogenetic analysis was that these different MGEs were lost from a SaPI5-negative USA300 NAE strain previously introduced into the facility, which was supported by an ancestral state reconstruction (Fig. 1 and Supplementary Fig. S1). Loss of SaPI5 is not an exceptional event in the USA300 lineage (approximately 7% of USA300 NA isolates according to Bianco *et al*. [12]) without it being known whether this excision confers any selective advantage. A maximum likelihood phylodynamic analysis performed using Treetime [17] indicated that the entry of this ancestral NAE population (already lacking SaPI5) in the long-term care hospital occurred in late 2018–early 2019, approximately 4 years before the current outbreak (Fig. 2). Of note, the estimate for the divergence of the NAE and SAE lineages herein (ca. 1963) is in line with that of Bianco *et al.* [12], between 1960 and 1970, supporting the validity of the present estimates. Finally, the SNPs matrix demonstrated that the isolate from the nurse (ST20230507) and those of two potential source patients for whom the nurse carried out daily dressings or assisted the doctor with an incision with drainage (ST20230844 [case 3], and ST20231016 [case 5]) exhibited a high degree of similarity (5 or 1 SNPs respectively, Supplementary Table S2), thereby favoring a scenario of direct transmission. To assess whether the loss of the MGEs affected fitness, the in vitro fitness (i.e. doubling time) of a selection of isolates was studied. The results showed a gradual increase in doubling time along with the sequential loss of the different MGEs (Supplementary Fig. S2).

We describe here a situation of likely transmission—although the full transmission chain was not identified—caused by a *S. aureus* USA300 strain with multiple MGEs excisions that occurred over a long dissemination period within a single institution. Although spontaneous loss of SCC*mec*IVa or ACME has been reported [11–13], the independent loss of several MGEs observed in the same care environment over a relatively short period of time is unique. Few studies have investigated in vitro fitness as a function of the presence/absence of MGEs in *S. aureus*. In a previous study using isogenic strains, we reported that the loss of SCCmec was associated with a shortened doubling time, whereas the loss of ACME led to an increased doubling time [10]. In the present series, alteration of the doubling time of MGE-deficient variants further supports the hypothesis that the USA300 genotype, which is disseminated on a global scale, is optimally adapted to a global context [3, 7]. The alteration of in vitro fitness associated with the loss of these MGEs may correspond to a transient step following a recent excision. We posit that epistatic interactions between MGEs and the core genome may contribute to these differences, although the worldwide success of the canonical clone likely stems from its broad ecological versatility rather than epistasis alone. More importantly, in the situation reported herein, it suggests that these MGEs were not required for epidemiological success, notably for inter-patient transmission that can be enhanced by several other factors. For instance, inter-patient physical contacts are frequent in this long-stay facility, where patient-residents meet and socialize in the communal areas provided for this purpose. In addition, although physically autonomous, their altered psychological state makes it difficult to impose barrier hygiene measures. Furthermore, antibiotic consumption is low thanks to the long-term antibiotic stewardship program implemented in this facility. Together, these conditions could have resulted in a low level of selection pressure, making virulence and resistance factors less essential for transmission and could have allowed genetic drift and niche specialization. The descriptive nature of our study and the limited understanding of MGE dynamics remain key limitations and call for further experimental work.

Nevertheless, this work illustrates that a relatively locked environment such as a long-term care hospital may constitute a quasi-enclosed habitat where genetic drift and niche specialization can rapidly occur. It is also of note that such a phenomenon has been observed with genome reduction in obligate intracellular pathogens [18] or flexibility in genomic island composition in *Escherichia coli*, which contributes to the adaptation of commensal and pathogenic *E. coli* [19].

Furthermore, this case study serves as a reminder that a preliminary screening prior to WGS based on phenotypic profiles or partial genomic profiles could result in a true epidemic being overlooked. In this instance, WGS enabled the connection of seemingly unrelated cases.

## Supplementary Material

Supplementary_material-revised_2b-accepted_ycaf105

## Data Availability

Read data of the 19 *S. aureus* isolates referred to the NRCS and of nine USA300 isolates from the NRCS collection are deposited on the European Nucleotide Archive (ENA): study accession number PRJEB82832. The 48 WGS data of previously published S. aureus strains are available in ref [12].
