# Supplementary material for: Gradual loss of mobile genetic elements in Staphylococcus aureus USA300 in a closed hospital niche
Source: ISME Commun. 2025 Jun 26;5(1):ycaf105. doi: 10.1093/ismeco/ycaf105 (PMC12265888; doi:10.1093/ismeco/ycaf105)
Supplement: Supplementary_material-revised_2b-accepted_ycaf105 [file supplementary_material-revised_2b-accepted_ycaf105.pdf]

Supplementary material for "*Gradual loss of mobile genetic element in Staphylococcus aureus USA300 in a closed hospital niche*"

Authors : AG Ranc, P. Martins Simões, B. Youenou, C. Kolenda, C. Dupieux-Chabert, F. Laurent, J.P. Rasigade, A. Tristan, F. Vandenesch

Supplementary Table S1: Clinical information on the 15 strains referred to the NRCS

Case	Strain number	Sex	Specimen date	Status	Sample site	Colonization/infection	Institution entry date	Typing
1	ST20230507	F	11/feb/2023	Nurse	Pulmonary	Necrotizing pneumonia	NA	ST8-MRSA
2	ST20230843<br>ST20231144	M	01/mar/2023<br>01/mar/2023	Patient	Nasal<br>Skin	Colonization<br>Recurrent furunculosis, multiple UTI	2019	ST8-MRSA
3	ST20230844	M	24/feb/2023	Patient	Skin	Recurrent furunculosis. Drained by case #1	2022	ST8-MRSA
4	ST20230845	F	01/mar/2023	Patient	Nasal	Colonization<br>Recurrent furunculosis	2018	ST8-MSSA
5	ST20231016	M	06/mar/2023	Patient	Skin	Abscess	2022	ST8-MRSA
6	ST20231146	F	11/apr/2023	Patient	Skin	Unknown	NA	ST398-MSSA
7	ST20231151	M	11/apr/2023	Patient	Nasal	Colonization<br>Recurrent furunculosis	2021	ST8-MRSA
8	ST20231152	F	12/apr/2023	Patient	Nasal	Colonization	NA	ST398-MSSA
9	ST20231153	F	11/apr/2023	Patient	Nasal	Colonization<br>Recurrent furunculosis	2019	ST8-MRSA
10	ST20231154	F	12/apr/2023	Patient	Nasal	Colonization	NA	ST5-MRSA
11	ST20231155<br>ST20231156	F	12/apr/2023<br>12/apr/2023	Patient	Nasal<br>Skin	Colonization<br>Unknown	NA	ST5-MRSA
12	ST20231157<br>ST20231158	M	04/apr/2023<br>12/apr/2023	Patient	Skin<br>Nasal	Recurrent furunculosis<br>Colonization	2022	ST8-MRSA<br>ST8-MRSA
13	ST20231159	F	12/apr/2023	Patient	Nasal	Colonization	2021	ST5-MSSA
	ST20231160		12/apr/2023		Skin	Colonization		ST8-MRSA
14	ST20231161	M	12/apr/2023	Patient	Nasal	Colonization	2021	ST8-MRSA
15	ST20231162	M	13/apr/2023	Patient	Nasal	Colonization	NA	ST398-MSSA

Supplementary Table S2: Single Nucleotides Polymorphism (SNPs) distance matrix and associated isolation dates

Strain	Isolation date	ST20230507	ST20230843	ST20230844	ST20230845	ST20231016	ST20231144	ST20231151	ST20231153	ST20231157	ST20231158	ST20231160	ST20231161
ST20230507	11/feb/2023	0	42	5	7	1	42	4	7	42	41	57	57
ST20230844	24/feb/2023	5	37	0	4	6	37	3	2	37	36	52	52
ST20231016	06/mar/2023	1	43	6	8	0	43	5	8	43	42	58	58
ST20231151	11/apr/2023	4	40	3	5	5	40	0	5	40	39	55	55
ST20231153	11/apr/2023	7	39	2	6	8	39	5	0	39	38	54	54
ST20230845	01/mar/2023	7	41	4	0	8	41	5	6	41	40	56	56
ST20230843	01/mar/2023	42	0	37	41	43	0	40	39	4	5	53	53
ST20231144	01/mar/2023	42	0	37	41	43	0	40	39	4	5	53	53
ST20231157	04/apr/2023	42	4	37	41	43	4	40	39	0	5	53	53
ST20231158	12/apr/2023	41	5	36	40	42	5	39	38	5	0	52	52
ST20231160	12/apr/2023	57	53	52	56	58	53	55	54	53	52	0	0
ST20231161	12/apr/2023	57	53	52	56	58	53	55	54	53	52	0	0

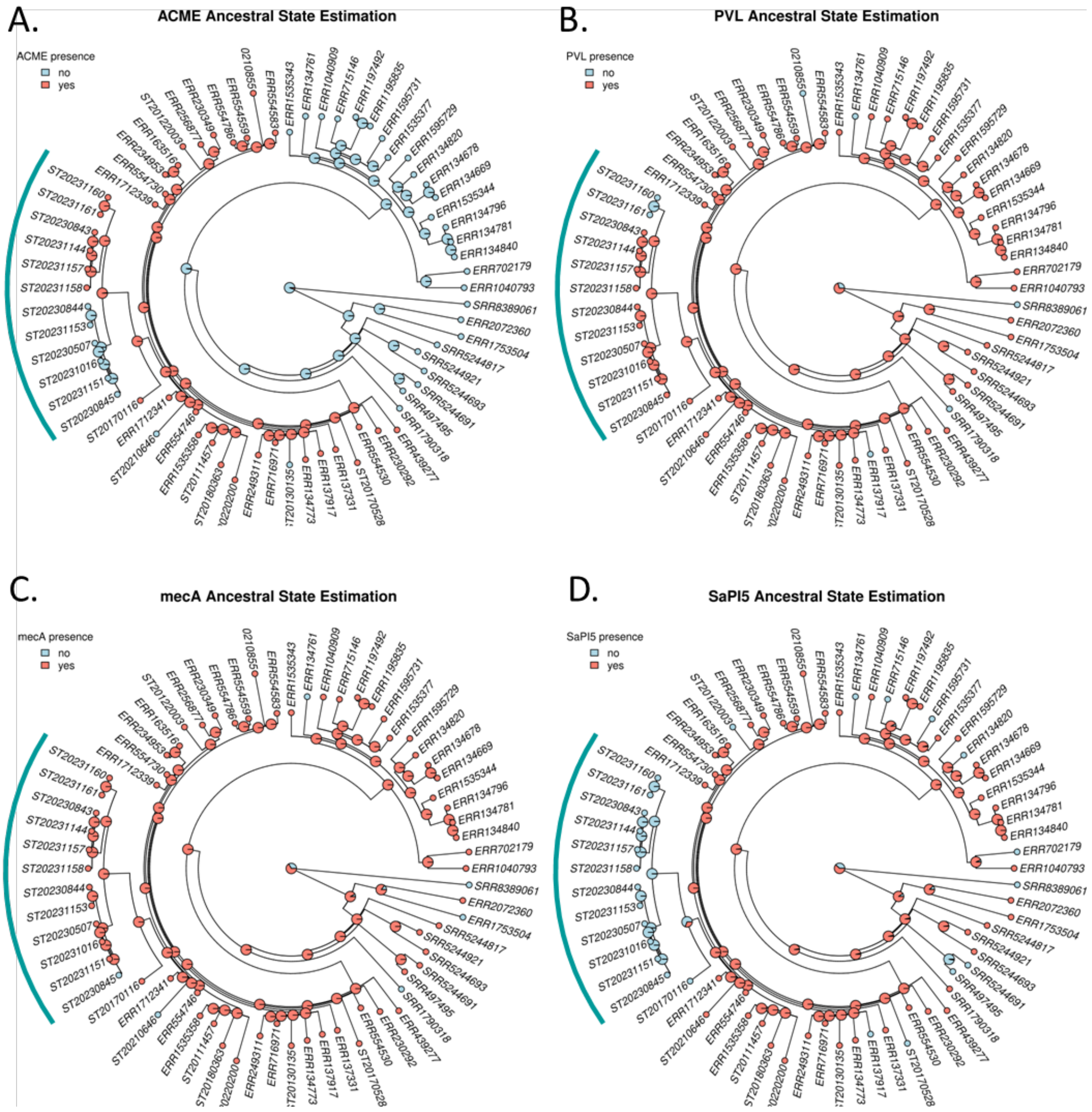

Supplementary Figure S1. Ancestral state reconstruction (ASR) of four mobile genetic elements (MGEs) in *Staphylococcus aureus* USA 300 strains.

Time-calibrated trees showing the presence (red) or absence (blue) of (A) ACME, (B) PVL phage, (C) *mec*A (SCC*mec*), and (D) SaPI5. The tree was time-calibrated using R package BactDating (https://doi.org/10.1093/nar/gky783) and ancestral states were reconstructed along the time-calibrated trees using maximum likelihood inference implemented in R package ape (https://doi.org/10.1093/bioinformatics/btg412).

Circles represent observed states at the tips and pie charts represent marginal posterior probabilities of ancestral states at internal nodes, based on a binary character model. Strain identifiers of strains associated with the epidemic clade are indicated by the blue arc on the side of the trees.

The basal node of the epidemic clade is inferred to be positive for *mec*A, ACME, and PVL, but negative for SaPI5. Loss of SaPI5 appears to have occurred multiple times independently across the tree, with no re-acquisition detected within the epidemic clade. Within the epidemic clade, two major subclades can be distinguished, one of which shows a complete loss of ACME. The *mec*A gene was lost once in the terminal isolates ST20230845, indicating recent, independent events. Additional *mec*A losses are observed in other parts of the tree, also on terminal branches. A pair of closely related isolates also share a common ancestor that lost PVL. These losses occurred on different internal nodes, suggesting diversifying selective pressures acting on the epidemic clade rather than a single event causing multiple MGE losses.

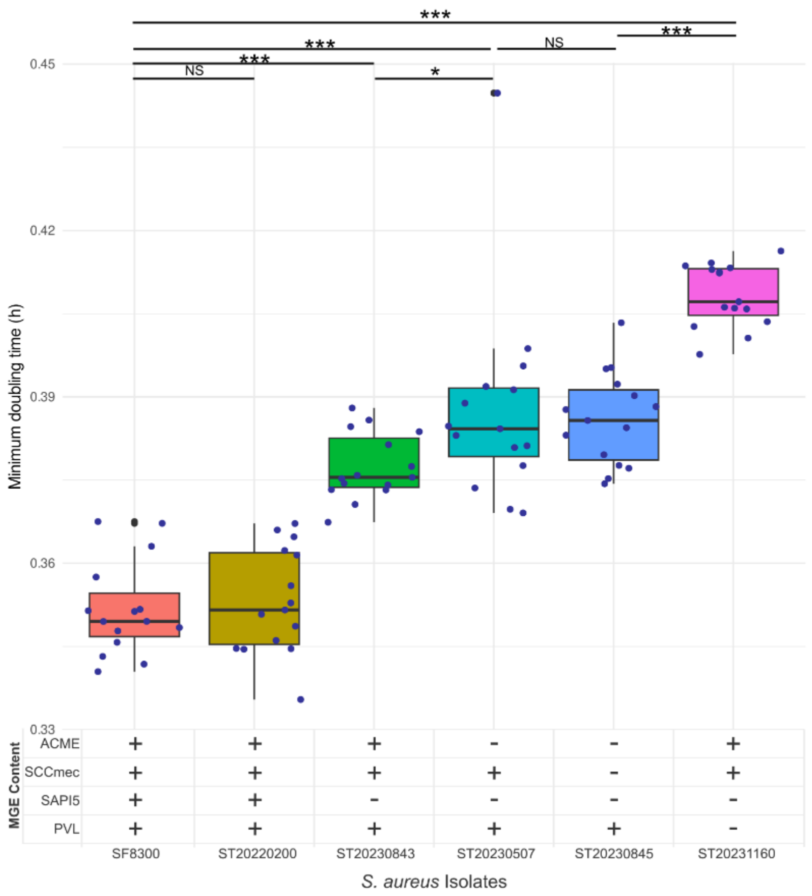

Supplementary Figure S2. Minimum doubling time (h) across different *Staphylococcus aureus* isolates categorized by MGE content.

Boxplot display the distribution of minimum doubling time values for each MGE content, with individual data points overlaid. Growth curves were determined from BHI cultures (initial Optical Density at 600 nm of 0.04) incubated in 96-well plates for 24 h at 37 °C with continuous optical density monitoring at 600 nm (Tecan Infinite® 200 PRO). Each isolate was inoculated in five independent wells (technical replicate), and the experiment was repeated on three different days (biological replicate). Minimum doubling time were obtained by analysing growth curve kinetics in R with the gcplyr package (https://doi.org/10.1186/s12859-024-05817-3).

A linear mixed-effects model (LMM) was used to assess the effect of MGE content of each isolate on minimum doubling time as a proxy of the fitness in laboratory condition. In this model, the experiment iteration was included as a random intercept to account for repeated measures across experiments (n = 15 observations, n = 3 experimental groups). Model estimation was performed using restricted maximum likelihood (REML) with the reference strain SF8300 as the baseline. The random effect of the experience iteration showed minimal variance ($\sigma^2 = 1.364 \times 10^{-5}$, SD = 0.0037), indicating negligible between-experiment variation. The fixed effects indicated significant differences in minimum doubling time based on MGE content for all isolates except ST20220200 that harbors the same MGE content as the reference strain. The ST20231160 isolate (PVL and SaPI5 negative isolate) exhibited the highest minimum doubling time (Estimate = 0.0566 ± 0.0036 hours, t = 15.89, $p < 0.001$), indicating a significantly slower growth rate. ST20230845 and ST20230507 (respectively ACME, SaPI5, SCC*mec* negative, and ACME, SaPI5 negative) also showed significantly higher minimum doubling times (Estimates = 0.0342 ± 0.0036 hours, t = 9.61 and 0.0359 ± 0.0036 hours, t = 10.09 respectively; both $p < 0.001$). ST20230843 (SaPI5 negative isolate) showed an intermediate effect (Estimate = 0.0256 hours ± 0.0036 hours, t = 7.20, $p < 0.001$). Model diagnostics (using DHARMa residual simulations package (https://cran.r-project.org/web/packages/DHARMa/vignettes/DHARMa.html), which generates standardized residuals by simulating response values from the fitted linear mixed-effect model, allowing for the assessment of model assumptions such as normality, homoscedasticity and independance) confirmed that residuals did not deviate from normality as (Kolmogorov-Smirnov test, $p > 0.05$), were not overdispersed (Dispersion test, $p > 0.05$), and variance was homogeneous across groups (Levene's test, $p > 0.05$).
